# Nulliparous Women’s Experience in the Immediate Postpartum Period After Cervical Ripening According to the Method: A Prospective Observational Study

**DOI:** 10.3390/jcm14072292

**Published:** 2025-03-27

**Authors:** Lea Delalandre, Lucie Planche, Guillaume Ducarme

**Affiliations:** 1 Department of Obstetrics and Gynecology, Centre Hospitalier Departemental, 85000 La Roche sur Yon, France; lea.delalandre@orange.fr; 2 Clinical Research Center, Centre Hospitalier Departemental, 85000 La Roche sur Yon, France; lucie.planche@ght85.fr

**Keywords:** cervical ripening, induction of labor, women’s experience, childbirth

## Abstract

**Background/Objectives:** Women’s experience plays an important role in the evaluation of different methods in obstetric practice with a patient-centered approach, especially for induction of labor (IOL). For IOL, cervical ripening seemed to be associated with a less positive experience of childbirth. More specific data regarding the different cervical ripening methods might help the obstetrician to better counsel and support women requiring cervical ripening to improve their experience of IOL. The aim of this study was therefore to assess whether the method is associated with altered experiences of cervical ripening and childbirth among nulliparous women. **Methods:** A prospective observational study included 340 nulliparous women with a singleton fetus in cephalic presentation and cervical ripening at term (≥37 weeks) for maternal and/or fetal disease using a cervical ripening balloon (CRB, 33.8%), dinoprostone vaginal insert (PG, 32.7%), oral misoprostol (M, 3.8%), or repeated methods (R, 29.7%). The cervical ripening method was left to the free discretion of the obstetrician responsible for the women. A self-developed questionnaire assessed women’s feelings and experience of cervical ripening and childbirth using Likert scales from 0 to 10 (very satisfied) according to the method in the immediate postpartum period. We compared the women’s experiences and feelings according to the cervical ripening method (CRB, PG, M, or R) and specifically studied the association (assessed by multivariate logistic regression analyses) between women’s experience in the immediate postpartum period and the cervical ripening method. **Results:** The overall vaginal rate was 79.1% and was similar between groups (CRB 77.4%, PG 87.4%, M 69.2%, and R 73.3%; *p* = 0.15). The experience of ripening was significantly better with CRB, M, or PG compared to R (CRB: 6.7 ± 2.5, PG: 7.2 ± 2.6, M: 6.8 ± 3.6, and R: 5.2 ± 2.8; *p* < 0.001). The maximum pain during ripening was significantly higher in PG (7.9 ± 2.5 vs. CRB 7.2 ± 2.4, M 7.0 ± 3.9, and R 7.8 ± 2.4; *p* = 0.02). The experience of childbirth was more negative in the R group (6.1 ± 2.7 vs. CRB 6.9 ± 2.6, PG 7.2 ± 2.4, and M 7.4 ± 3.1; *p* = 0.02). After multivariate analysis with adjustment for confounders (method for cervical ripening, pain during IOL, mode of birth, maternal morbidity, and neonatal morbidity), repeated methods were significantly associated with worse overall experiences of cervical ripening (aOR = −1.4, 95%CI −2.1–−0.67; *p* < 0.001) and childbirth (aOR = −0.75, 95% CI −1.6–−0.05; *p* = 0.03), compared to PG, CRB, or M used alone. After adjustment, maternal experience and childbirth were similar between methods used alone for cervical ripening. **Conclusions:** Nulliparous women who required repeated methods for cervical ripening at term had significantly worse experiences of ripening and childbirth in the immediate postpartum period compared to PG, CRB, or M used alone, regardless of the mode of delivery and maternal and neonatal morbidity.

## 1. Introduction

Induction of labor (IOL) has steadily increased in most countries during the last decade, now initiating more than 30% of all childbirths [1,2]. Although much data comparing different methods are available [3,4,5], there are insufficient comparative data to determine which of the methods has the most effective safety profile with the best women’s experience and overall satisfaction of childbirth. Variation in parity, gestational age, and indications for IOL in clinical practice, especially in the case of repeated methods for IOL, as well as other demographic features, undoubtedly also affects clinical results. Variations may also lead to an important delay between the onset of cervical ripening, the onset of labor, and the birth that may depreciate women’s well-being and satisfaction. Moreover, positive experiences of childbirth and maternal and neonatal outcomes are important to women [6] and are known to influence physical, social, and psychological short-term and long-term outcomes [7].

Women’s experience plays an important role in the evaluation of different methods in obstetric practice with a patient-centered approach [8,9,10]. Women’s experiences of childbirth and satisfaction differ depending on the type of labor (spontaneous or induced), the mode of delivery (vaginal, cesarean) and parity and are controversial [6,11,12,13]. For IOL, cervical ripening seemed to be associated with a less positive experience of childbirth, only partly explained by interventions and complications of delivery [14,15]. Regarding cervical ripening, there are some data about women’s experiences according to the methods [15,16,17,18,19,20].

More specific data regarding the different cervical ripening methods might help the obstetrician to better counsel and support women requiring cervical ripening to improve their experience of IOL. The aim of this study was to compare the immediate postpartum feelings and experiences of women who required cervical ripening at term (≥37 weeks) for maternal and/or fetal disease using a cervical ripening balloon (CRB), dinoprostone vaginal insert (PG), oral misoprostol (M), or repeated methods (R).

## 2. Materials and Methods

This was a planned secondary analysis of a prospective cohort study that included all consecutive women aged ≥18 years with live fetuses at term (≥37 weeks) who required cervical ripening (Bishop score at enrollment < 6) for maternal and/or fetal indication using PG, CRB, oral misoprostol (M), or repeated methods (R) from 9 January 2020 to 30 June 2021 in a tertiary care university hospital [21]. For this study, only nulliparous women with a singleton in cephalic position were included. Exclusion criteria were multiple pregnancy, history of uterine scar, non-cephalic presentation, active or purulent infection of the lower genital tract, fetus with suspected severe congenital abnormalities, contraindication to prostaglandins, allergy to latex, non-reassuring cardiotocograph (CTG) before cervical ripening, and inability to speak and read French. Women who chose not to participate after receiving information were also excluded.

This present study was conducted in accordance with the French-approved guidelines. Eligible women were approached by their local caregiver immediately before IOL. All participants received written information about the study using institutional review board-approved documents. Written consent is not required for prospective observational study according to the French law, but each woman had the opportunity to opt out of the analysis. After giving written information and oral consent, an anonymous number was allocated to each included woman. The study protocol was approved by a Research Ethics Committee (Groupe Nantais d’Ethique dans le Domaine de la Santé (GNED), n° 2020-01-08) on 8 January 2020 before the beginning of the study.

As previously described [21,22,23], all included women were admitted to the labor ward and recorded at least 30 min of normal CTG before (and after) the cervical ripening method. CRB (Cervical Ripening Balloon^®^, Cook OB/GYN, Spencer, IN, USA) and dinoprostone vaginal insert with 10 mg slow-release dinoprostone (PG, Propess^®^, Ferring, Saint-Prex, Switzerland) are approved for use up to 24 h and were removed before 24 h in case of spontaneous labor, expulsion, non-reassuring CTG result, spontaneous rupture of membranes, or unexplained vaginal bleeding. Oral misoprostol (M, Angusta^®^, Azanta, Valby, Denmark) was used for more than 24 h (200 µg per day). In our center, the cervical ripening method was left to the free discretion of the obstetrician responsible for the woman at the beginning of cervical ripening [21]. If labor does not ensue or the Bishop score is still unfavorable (score < 6) after 24 h, there is no consensus, and repeating the method for cervical ripening is possible at the free discretion of the treating physician. Once the Bishop score was ≥6, further management with amniotomy and oxytocin induction was recommended by our team [21].

As previously detailed [21], all data about women’s medical history, information regarding course of the pregnancy, cervical ripening (indication, method and cervical status before and after cervical ripening), mode (oxytocin induction, amniotomy) and duration (from 3-cm to delivery) of labor, mode of anesthesia (epidural or not, time of fitting the epidural anesthesia catheter), mode of delivery (spontaneous or operative vaginal delivery, cesarean section), gestational age at birth, standard perinatal outcomes, maternal and neonatal immediate postpartum period, and immediate women’s experience and satisfaction were collected from a prospectively maintained database of women who were included throughout the study period.

Indications for cervical ripening consisted of prolonged pregnancy, prelabor rupture of the membranes, hypertensive disorders of pregnancy, fetal growth restriction (FGR), abnormality of fetal vitality (oligoamnios or decreased fetal movements before 41 weeks), gestational or previous diabetes, macrosomia without diabetes, and other maternal and/or fetal indications, as previously defined in the published study protocol [21].

As previously described [21], maternal outcomes included severity of perineal tears, postpartum hemorrhage (PPH, defined as bleeding 500 mL or greater), need for sulprostone, second-line therapies (Bakri balloon, uterine compression sutures, uterine artery embolization, and peripartum hysterectomy) for management of persistent PPH, chorioamnionitis, infections (defined by at least one of the following: endometritis, episiotomy infection, or wound infection requiring surgery), blood transfusion, intensive care unit admission, maternal stay, and maternal death. Maternal morbidity was defined by at least one of the following criteria: chorioamnionitis, third- or fourth-degree perineal tears, PPH, need for additional uterotonic agents, second-line therapies, blood transfusion, admission to the intensive care unit, and maternal death.

Neonatal immediate outcomes included shoulder dystocia, umbilical arterial blood gases at birth, 5-min Apgar score, respiratory distress syndrome, phototherapy for neonatal jaundice, intraventricular hemorrhage, need for resuscitation or intubation, any transfer to the neonatal intensive care unit (NICU) for close monitoring of the neonate, sepsis (defined as confirmed clinical infection with positive bacteriological tests), seizures, and neonatal death [21]. Neonatal morbidity was defined by at least one of the following criteria: 5 min Apgar score of less than 7, pH of less than 7.10, shoulder dystocia, need for resuscitation or intubation, intraventricular hemorrhage, respiratory distress syndrome, hyperbilirubinemia, sepsis, seizures, an NICU admission longer than 24 h, and neonatal death.

Included women were invited to fill in the questionnaire only once, blinded from the obstetrical staff, during the immediate postpartum period (1–4 days after birth) before discharge. The questionnaire was self-developed and consisted of questions about women’s current feelings and preference for management of cervical ripening. First, women were asked to indicate the discomfort/pain and experience during the insertion of CRB or PG and while the system was in situ. The levels of discomfort/pain assessments were rated on a visual analog scale of 1–10. Second, we asked women to indicate their feelings about the duration of IOL. Women were asked to answer this question on a 10-point Likert-like scale (“0 = too long and unbearable” to “10 = acceptable”). The specific experience about IOL and the women’s overall satisfaction regarding IOL, labor, and delivery were also explored with a 10-point Likert-like scale (“0 = very dissatisfied” to “10 = very satisfied”). Finally, we asked women whether they would prefer the same method again in a future pregnancy that required cervical ripening (Supplementary File S1).

The primary endpoint was the women’s overall satisfaction for cervical ripening. The secondary endpoints were the women’s overall experience of childbirth, vaginal discomfort, pain and experience during cervical ripening, feelings about the duration of IOL, and the preferred method for cervical ripening in a future pregnancy.

Continuous data were described by their means ± standard deviations and compared by *t*-tests (or Mann–Whitney tests when appropriate), and categorical data were described by percentages and compared by chi-square tests (or Fisher exact tests when appropriate). As previously reported [17,24,25], poor experience of childbirth was defined as a scale score < 5. We compared the women’s experiences and feelings according to the cervical ripening method (CRB, PG, M, or R) and specifically studied the association (assessed by multivariate logistic regression analyses) between women’s experiences in the immediate postpartum period and cervical ripening method. The multivariate logistic regression allowed us to analyze (together) the effect of other risk factors and potential confounders (method for cervical ripening, pain during IOL, mode of birth, maternal morbidity, and neonatal morbidity). No formal sample size was calculated as we collected data for all cases over a specified time period. We used R software (version 4.2.0 Patched) for all the analyses. A *p* value < 0.05 was considered statistically significant.

## 3. Results

**Figure 1:**
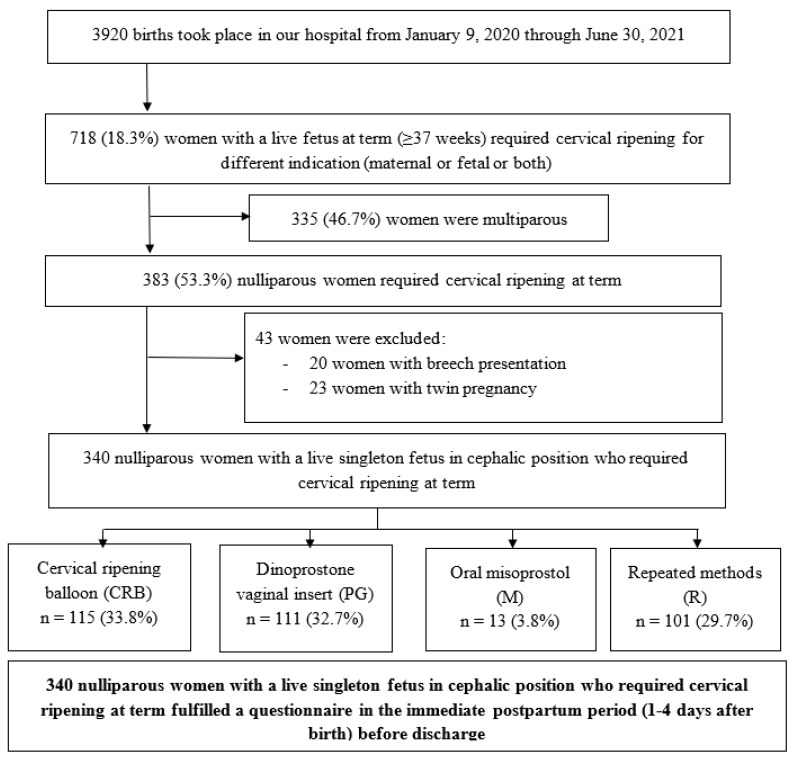
Flow chart.

During the study period, 3920 births and 502 cesarean deliveries (12.8%) were recorded in our tertiary public hospital, and 718 women (18.3%) with a live fetus at term required cervical ripening for different indications (maternal or fetal or both) in our hospital. Among these women, 340 were nulliparous women and were finally included in this study (Figure 1).

The maternal characteristics, according to the method, are shown in Table 1.

Among 340 included nulliparous women, 115 cervical ripening balloons (CRB, 33.8%), 111 dinoprostone vaginal inserts (PG, 32.7%), 13 oral misoprostol (M, 3.8%), and 101 repeated methods (R, 29.7%) were used for cervical ripening at term, at a mean gestational age of 39.9 ± 1.4 weeks (Table 1). Indication for cervical ripening significantly differed between groups. Among women with diabetes (GDM or previous), PG was used less frequently than the other methods, and among women with other medical indications (i.e., thrombocytopenia, intrahepatic cholestasis of pregnancy, hydramnios, other maternal complications), CRB was used less frequently than the other methods (Table 1). The rate of favorable cervix (Bishop score > 6) within 24 h did not differ between CRB, PG, or M. Nevertheless, time from device insertion to delivery and mode of labor significantly differed according to the method, with shorter time from device insertion to delivery and more labor after cervical ripening using PG or M compared to CRB (Table 1). The epidural anesthesia catheter was placed at different times during labor according to the women’s choice, and these times were not significantly different between groups.

The overall vaginal rate was 79.1% and was similar between groups (CRB 77.4%, PG 87.4%, M 69.2%, and R 73.3%; *p* = 0.15). Compared to the overall vaginal delivery rate during the study period in our hospital (87.2%), the rate of vaginal delivery in the study (79.1%) was significantly lower (*p* < 0.001). Immediate maternal outcomes and maternal morbidity did not differ according to the method. Repeated methods for cervical ripening seemed not to be associated with higher maternal morbidity, compared to other methods (Table 1). Neonatal immediate outcomes and neonatal morbidity did not differ according to the method used for cervical ripening (Table 2).

All included women in this study had completed the questionnaire during the study period. Maximal pain during ripening, the moment of maximal pain during ripening, vaginal discomfort due to the device, feelings about the duration of IOL, and the preferred method for cervical ripening in a future pregnancy significantly differed according to the method (Table 3).

The women’s experience of cervical ripening was significantly better with CRB, M, or PG compared to R (CRB: 6.7 ± 2.5, PG: 7.2 ± 2.6, M: 6.8 ± 3.6, and R: 5.2 ± 2.8; *p* < 0.001). The maximal pain during ripening was significantly higher in the PG group (7.9 ± 2.5 vs. CRB 7.2 ± 2.4, M 7.0 ± 3.9, and R 7.8 ± 2.4; *p* = 0.02). The overall experience of childbirth was more negative in the R group (6.1 ± 2.7 vs. CRB 6.9 ± 2.6, PG 7.2 ± 2.4, and M 7.4 ± 3.1; *p* = 0.02) (Table 3).

The women’s experience of cervical ripening and childbirth significantly differed according to the mode of birth (spontaneous vaginal, operative vaginal, or cesarean delivery), with a worse experience of cervical ripening and childbirth after cesarean delivery compared to spontaneous vaginal or operative vaginal delivery (4.7 ± 3.0 vs. 7.0 ± 2.5 vs. 6.5 ± 2.7; *p* < 0.001 and 5.2 ± 3.2 vs. 7.4 ± 2.2 vs. 6.5 ± 2.4; *p* < 0.001, respectively). Neonatal morbidity also altered significantly the women’s experience of cervical ripening and childbirth (5.6 ± 3.0 vs. 6.7 ± 2.7; *p* = 0.006 and 6.2 ± 2.7 vs. 7.0 ± 2.6; *p* = 0.010, respectively).

After multivariate analysis with adjustment for confounding variables (method for cervical ripening, pain during IOL, mode of birth, maternal morbidity, and neonatal morbidity), repeated methods (R) were significantly associated with a worse experience of cervical ripening (aOR = −1.4, 95%CI −2.1–−0.67; *p* < 0.001) and a worse overall experience of childbirth (aOR = −0.75, 95%CI −1.6–−0.05; *p* = 0.03), compared to PG, CRB, or M used alone. After adjustment, maternal experiences of cervical ripening and childbirth were similar between methods used alone (Table 4).

## 4. Discussion

In our study, nulliparous women who required repeated cervical ripening methods at term had a significantly worse experience of cervical ripening and childbirth in the immediate postpartum period compared to women who had cervical ripening balloons, dinoprostone vaginal inserts, or misoprostol alone, regardless of the mode of delivery and maternal and neonatal morbidity.

Much data about women’s experiences and feelings about IOL, cervical ripening, and childbirth are available. First, IOL for maternal and/or fetal indication is associated with a poorer birth experience compared to spontaneous labor, and women often expressed a desire to avoid IOL [12,26]. A recent prospective study of 2042 women (575 women with IOL and 1467 with spontaneous-onset labor) showed that IOL was a risk factor of dissatisfaction regarding pain relief and overall birth experience, compared to spontaneous-onset labor. Poor pain relief, its incorrect timing, and deficient information on pain relief were strong predictive factors of dissatisfaction with the overall birth experience [12]. Second, cervical ripening seems to be associated with a poorer women’s experience compared to IOL with a favorable cervix and oxytocin. Data from a French prospective population-based cohort (MEDIP), including 3042 consecutive women with IOL and a live fetus during one month in seven perinatal networks, showed that cervical ripening seemed to be associated with a less positive experience of childbirth at 2 months postpartum, compared to oxytocin [15]. Compared with oxytocin (*n* = 541), cervical ripening (*n* = 910) was associated less often with feelings that labor went ‘as expected’, length of labor was ‘acceptable’, ‘vaginal discomfort’ was absent, and with lower global satisfaction (aOR = 0.90, 95% CI 0.84–0.96). Interventions and maternal and neonatal complications mediated between 6 and 35% of the total effect of cervical ripening on maternal experience [15]. In the literature, several predictors of poor childbirth experience with labor induction have been reported. Specifically, information provided, long labor, cesarean section, and PPH were often associated with a negative childbirth experience in induced labor [6,13,24,25,26,27,28]. We reported a significantly higher rate of cesarean delivery in our study compared to the rate of cesarean delivery in our hospital during the study period (20.9% vs. 12.8%, *p* < 0.001). That may be easily explained by the fact that all included women in the study required cervical ripening for maternal and/or fetal indication, and these pregnancies may then be considered as high-risk pregnancies for obstetric complications. That is why we have decided to analyze women’s experiences and feelings using multivariate analysis with adjustment for mode of delivery and maternal and neonatal morbidity, even though no difference was observed in univariate analysis between groups. Third, published data about different methods for cervical ripening (PG, CRB, and M) reported variable results about women’s feelings [15,16,17,18,19,20]. Our study did not show any difference in maternal experience according to the methods used for cervical ripening. In the MEDIP cohort, as in our study, compared to PG, maternal experience was not significantly different with the other prostaglandins (M), and CRB was associated with less pain [15]. A secondary analysis of an RCT among 502 women undergoing IOL at term with M or Foley catheters reported similar experiences of the duration of labor, pain during labor, general satisfaction with labor, and feelings of control and fear related to their expectation using a questionnaire within 24 h after delivery [16]. More recently, a retrospective study including 365 women after cervical ripening that was left to the patient’s free appreciation (M (*n* = 129, 35.3%) or CRB (*n* = 236, 64.7%)) reported that satisfaction is overall good, irrespective of the method when women choose the method of cervical ripening [17]. Nevertheless, a recent before-and-after comparative study including all women undergoing cervical ripening compared two separate two-month periods (the first period with PG (*n* = 81) and the second period used M (*n* = 81)). Oral misoprostol was associated with higher levels of satisfaction and reduced discomfort compared to PG, assessed using the EXIT questionnaire [20].

Our study presents several strengths. First, the study was a planned secondary analysis of data collected for a single-center prospective observational study. Second, the strengths of this study lie in the homogeneous nature of the population studied in a single center (only consecutive women with a singleton fetus in cephalic position who had cervical ripening at term), which avoided significant variations regarding cervical ripening and delivery management, and all women were evaluated in the immediate postpartum period using the same questionnaire. Third, another strength of the study is the use of multiple aspects for evaluating the women’s experiences and feelings of cervical ripening and childbirth and a statistical analysis that accounted for multiple confounding factors including mode of delivery, birthweight, maternal morbidity, and neonatal morbidity. Our results must be interpreted in light of certain limitations. First, the main limitation of this study is that our study reflects the experience of one tertiary hospital, and the results may not be generalizable to all maternity wards using different methods for cervical ripening. Specifically, we only included women who required cervical ripening for maternal and/or fetal indication, and the cervical ripening method was left to the free discretion of the obstetrician responsible for the woman at the beginning of cervical ripening. It may be interesting to compare our results with a prospective cohort of women who have chosen cervical ripening at term without any indication and who have chosen the method. Second, no sample size to demonstrate the feasibility of the study was calculated before the study. Third, although the sample size of this prospective study was important (*n* = 340), our study may lack sufficient statistical power to detect small but clinically relevant differences between methods in women’s experiences registered in the immediate postpartum period. Fourth, immediate postpartum evaluation of childbirth experience may be altered by maternal and/or neonatal complications, and it may be interesting to assess experience of childbirth in the long term. This study was continued with an evaluation of childbirth experience at 3 months [21]; the results were not yet published.

## 5. Conclusions

Nulliparous women who required repeated cervical ripening methods at term had a significantly worse experience and feelings of cervical ripening and childbirth in the immediate postpartum period compared to women who had PG, CRB, or M alone, regardless of the mode of delivery and maternal and neonatal morbidity. The study might help the obstetrician to better counsel, provide adapted information, and support women requiring cervical ripening to improve their experience of IOL according to the method, and, specifically, repeated methods for cervical ripening might be questioned.

## Figures and Tables

**Table 1:** Maternal and labor characteristics and maternal outcome according to the method used for cervical ripening (*n* = 340).

	Cervical Ripening Balloon, *n* = 115 (33.8%)	Dinoprostone Vaginal Insert, *n* = 111 (32.7%)	Oral Misoprostol, *n* = 13 (3.8%)	Repeated Method, *n* = 101 (29.7%)	*p* Value
Age, years	28.1 ± 5.7	28.3 ± 4.8	30.0 ± 5.9	29.3 ± 4.7	0.22
Pre-pregnancy BMI, kg/m^2^	26.1 ± 6.2	24.1 ± 4.0	23.7 ± 4.3	28.2 ± 7.0	0.24
Obesity (BMI ≥ 30 kg/m^2^)	30 (26.1)	11 (9.9)	1 (7.7)	37 (36.6)	<0.001
Tobacco use	14 (12.2)	23 (20.7)	2 (15.4)	18 (17.8)	0.41
Previous diabetes	4 (3.5)	1 (0.9)	0	1 (1.0)	0.50
Chronic hypertension	0	0	1 (7.7)	1 (1.0)	0.02
Gestational age at cervical ripening, weeks	39.9 ± 1.6	40.0 ± 1.3	40.0 ± 1.0	39.2 ± 1.4	0.43
Indication for cervical ripening					
Prolonged pregnancy	33 (28.7)	33 (29.7)	2 (15.4)	18 (17.8)	0.06
Pregnancy-associated hypertensive disorders	1 (0.9)	0	0	1 (1.0)	0.94
Fetal growth restriction	9 (7.8)	0	0	6 (5.9)	0.60
Diabetes (GDM or previous)	31 (27.0)	10 (9.0)	3 (23.1)	16 (15.8)	<0.001
Antenatal suspicion of macrosomia without diabetes	5 (4.3)	3 (2.7)	0	3 (3.0)	0.62
Abnormality of fetal vitality	8 (7.0)	0	0	5 (5.0)	0.56
Other medical indication	28 (24.3)	65 (58.6)	8 (61.5)	52 (51.5)	<0.001
Bishop score < 3 before cervical ripening	39 (33.9)	24 (21.6)	3 (23.1)	43 (42.6)	0.01
Uterine tachysystole	0	2 (1.8)	0	2 (2.0)	0.50
Favorable cervix (Bishop score > 6) within 24 h	106 (92.2)	97 (87.4)	12 (92.3)	0	<0.001
Mode of labor					<0.01
Labor after cervical ripening	44 (38.3)	98 (88.3)	9 (69.2)	54 (53.5)	
Artificial rupture of membranes and oxytocin	68 (59.1)	13 (11.7)	2 (15.4)	41 (10.6)	
Cesarean delivery for cervical ripening failure	3 (2.6)	0	2 (15.4)	6 (5.9)	
Gestational age at birth, weeks	40.1 ± 1.4	40.3 ± 1.3	40.3 ± 1.1	39.9 ± 1.5	0.42
Mode of delivery					0.15
Spontaneous vaginal delivery	65 (56.5)	73 (65.8)	8 (61.5)	58 (57.4)	
Operative vaginal delivery	24 (20.9)	24 (21.6)	1 (7.7)	16 (15.9)	
Cesarean delivery during labor	23 (20.0)	14 (12.6)	2 (15.4)	21 (20.8)	
Epidural analgesia	110 (95.7)	108 (97.3)	13 (100)	91 (90.1)	0.91
Duration of labor (3-cm to delivery)	8.4 ± 4.6	7.2 ± 4.2	7.0 ± 4.9	8.1 ± 5.5	0.23
Time from device insertion to delivery	32 ± 9	19 ± 10	17 ± 9	49 ± 42	<0.001
PPH	12 (10.4)	11 (9.9)	1 (7.7)	5 (5.0)	0.45
Episiotomy	40 (24.8)	34 (30.6)	1 (7.7)	26 (25.7)	0.22
Third- or fourth-degree perineal	6 (5.2)	10 (9.0)	1 (7.7)	5 (5.0)	0.24
Need for additional uterotonic agent (sulprostone)	3 (2.6)	8 (7.2)	0	2 (2.0)	0.21
Second-line therapies	5 (4.3)	7 (6.3)	0	2 (2.0)	0.47
Chorioamnionitis	0	3 (2.7)	0	2 (2.0)	0.43
Infections	0	1 (0.9)	0	0	0.77
Blood transfusion	0	3 (2.7)	0	0	0.22
Intensive care unit admission	0	0	0	0	-
Maternal stay, days	4.7 ± 1.6	4.5 ± 1.8	4.2 ± 0.9	4.8 ± 1.9	0.09
Maternal death	0	0	0	0	-
Maternal morbidity *	18 (15.7)	27 (24.3)	2 (15.4)	13 (12.9)	0.15

Values are given as mean ± SD or number (percentage) unless otherwise indicated. BMI, body mass index; GDM, gestational diabetes mellitus; ART, assisted reproductive technology; SGA, small-for-gestational-age; PPH, postpartum hemorrhage; IQR, interquartile range. Other medical indications were defined as cervical ripening due to thrombocytopenia, cholestasis in pregnancy, hydramnios, or other maternal disease. Severe PPH was defined as bleeding 1000 mL or greater. Second-line therapies were defined as Bakri balloon, uterine compression sutures, uterine artery embolization, and peripartum hysterectomy for management of massive persistent PPH after failure of uterine massage and uterotonic agents to stop bleeding. Infections were defined by at least one of the following: endometritis, episiotomy infection, or wound infection requiring surgery. * Maternal morbidity was defined by at least one of the following criteria: chorioamnionitis, third- or fourth-degree perineal tears, PPH, need for additional uterotonic agents, second-line therapies, blood transfusion, admission to the intensive care unit, and maternal death.

**Table 2:** Neonatal outcome according to the method used for cervical ripening (*n* = 340).

	Cervical Ripening Balloon, *n* = 115 (33.8%)	Dinoprostone Vaginal Insert, *n* = 111 (32.7%)	Oral Misoprostol, *n* = 13 (3.8%)	Repeated Method, *n* = 101 (29.7%)	*p* Value
Gestational age at birth, weeks	40.1 ± 1.4	40.3 ± 1.3	40.3 ± 1.1	39.9 ± 1.5	0.42
Birth weight, g	3272 ± 527	3427 ± 499	3115 ± 401	3278 ± 529	0.21
5-min Apgar score of less than 7	3 (2.6)	2 (1.8)	0	4 (4.0)	0.74
pH of less than 7.10	4 (3.5)	9 (8.1)	0	5 (5.0)	0.47
Meconium-stained amniotic fluid	0	2 (1.8)	0	0	0.40
Chorioamnionitis	0	3 (2.7)	0	2 (2.0)	0.43
Shoulder dystocia	5 (4.3)	8 (7.2)	0	4 (4.0)	0.70
Need for resuscitation or intubation	9 (7.8)	14 (12.6)	1 (7.7)	7 (6.9)	0.58
Respiratory distress syndrome	11 (9.6)	17 (15.3)	1 (7.7)	13 (12.9)	0.63
Neonatal jaundice	3 (2.6)	4 (3.6)	1 (7.7)	1 (1.0)	0.32
Sepsis	0	3 (2.7)	0	0	0.22
Seizures	0	0	0	0	-
Intraventricular hemorrhage greater than grade 2	0	1 (0.9)	0	0	0.77
NICU admission	11 (9.6)	8 (7.2)	0	11 (10.9)	0.73
Neonatal death	0	0	0	0	-
Neonatal morbidity *	24 (20.9)	31 (27.9)	2 (15.4)	26 (25.7)	0.65

Values are given as mean ± SD or number (percentage) unless otherwise indicated. NICU, neonatal intensive care unit. * Neonatal morbidity was a composite variable, defined by at least one of the following criteria: 5 min Apgar score of less than 7, pH of less than 7.10, shoulder dystocia, need for resuscitation or intubation, intraventricular hemorrhage, respiratory distress syndrome, hyperbilirubinemia, sepsis, seizures, an NICU admission longer than 24 h, and neonatal death.

**Table 3:** Questionnaire in the immediate postpartum period according to the method used for cervical ripening.

	Cervical Ripening Balloon, *n* = 115 (33.8%)	Dinoprostone Vaginal Insert, *n* = 111 (32.7%)	Oral Misoprostol, *n* = 13 (3.8%)	Repeated Method, *n* = 101 (29.7%)	*p* Value
Maximal pain during ripening	7.2 ± 2.4	7.9 ± 2.5	7.0 ± 3.9	7.8 ± 2.4	0.02
Moment of maximal pain during ripening					<0.001
No pain	8 (7.0)	13 (11.7)	13 (100)	11 (10.9)	
Only at insertion	52 (45.2)	18 (16.2)	-	49 (48.5)	
While the device was in vagina	55 (47.8)	79 (71.1)	-	40 (39.6)	
Vaginal discomfort					<0.001
0/10	11 (9.6)	55 (49.5)	-	14 (13.9)	
≥5/10	71 (61.7)	33 (29.7)	-	60 (59.4)	
Feelings about the duration of IOL	6.3 ± 3.1	6.9 ± 3.1	6.5 ± 4.0	4.3 ± 3.4	<0.001
Women’s experience for ripening	6.7 ± 2.5	7.2 ± 2.6	6.8 ± 3.6	5.2 ± 2.8	<0.001
Preferred method for ripening in a future pregnancy	79 (68.7)	78 (70.3)	8 (61.5)	46 (45.5)	0.001
Women’s experience of childbirth	6.9 ± 2.6	7.2 ± 2.4	7.4 ± 3.1	6.1 ± 2.7	0.02

Values are given as mean ± SD or number (percentage) unless otherwise indicated. IOL, induction of labor.

**Table 4:** Multivariate analysis of women’s satisfaction for induction of labor and childbirth after cervical ripening.

	Women’s Satisfaction of Childbirth		Women’s Satisfaction of Cervical Ripening	
	Adjusted OR (95% CI)	*p*-Value	Adjusted OR (95% CI)	*p*-Value
Methods		0.03		<0.001
CRB	Reference		Reference	
M	0.56 [−0.89–2.0]		0.29 [−1.3–1.8]	
PG	0.22 [−0.48–0.86]		0.49 [−0.21–1.42]	
R	−0.75 [−1.6–−0.05]		−1.4 [−2.1–−0.67]	
Mode of birth		<0.001		<0.001
Cesarean delivery	Reference		Reference	
Operative vaginal delivery	1.5 [0.52–2.40]		1.8 [0.78–2.51]	
Spontaneous vaginal delivery	2.0 [1.42–2.71]		1.7 [1.22–2.63]	
Maternal morbidity ^1^		0.09		0.40
Yes	Reference		Reference	
No	0.60 [−0.10–1.3]		0.29 [−0.44–1.0]	
Neonatal morbidity ^2^		0.09		0.01
Yes	Reference		Reference	
No	0.53 [−0.08–1.1]		0.88 [0.24–1.5]	

OR, odds ratio; CI, confidence interval; CRB, cervical ripening balloon; M, oral misoprostol; PG, vaginal dinoprostone insert; R, repeated methods. ^1^ Maternal morbidity was defined by at least one of the following criteria: chorioamnionitis, third- or fourth-degree perineal tears, PPH, need for additional uterotonic agents, second-line therapies, blood transfusion, admission to the intensive care unit, and maternal death. ^2^ Neonatal morbidity was defined by at least one of the following criteria: 5 min Apgar score of less than 7, pH of less than 7.10, shoulder dystocia, need for resuscitation or intubation, intraventricular hemorrhage, respiratory distress syndrome, hyperbilirubinemia, sepsis, seizures, an NICU admission longer than 24 h, and neonatal death.

## Data Availability

The data presented in this study are available on request from the corresponding author. The data are not publicly available due to institutional policy.

## Supplementary Materials

The following supporting information can be downloaded at: https://www.mdpi.com/article/10.3390/jcm14072292/s1, File S1: A self-developed questionnaire assessed women’s feelings and experience of cervical ripening and childbirth.
