# Supplementary material for: Nulliparous Women’s Experience in the Immediate Postpartum Period After Cervical Ripening According to the Method: A Prospective Observational Study
Source: J Clin Med. 2025 Mar 27;14(7):2292. doi: 10.3390/jcm14072292 (PMC11989454; doi:10.3390/jcm14072292)
Supplement: Supplementary file 1 [file jcm-14-02292-s001.zip › jcm-3502713-supplementary.pdf]

QUESTIONNAIRE MATUCOL

This questionnaire aims to collect your experience of induction of labor (= period that begins with the use of a device to modify your cervix (=cervical ripening) and ends as soon as you are in labor). This questionnaire is anonymous. Thank you in advance for your participation.

Date : ... /... / …

Maximum pain during cervical ripening? …………………………………

(On a visual analog scale 1-10, “0=no pain” to “10=maximum pain”)

Complete these 2 questions if a vaginal device was used for the cervical ripening. Thank you.

What was the most painful moment for you?

❐ No pain ❐ Only during the insertion of the device ❐ When the device was in situ

Vaginal discomfort during cervical ripening? …………………………………

(On a 10-point Likert-like scale, “0=no vaginal discomfort” to “10=maximum vaginal discomfort”)

What do you think about the duration of induction of labor? ………………………………

(On a visual analog scale 1-10, “0=too long and unbearable” to “10=acceptable”)

Overall, how do you feel about your experience of induction of labor? ……………..

(On a visual analog scale 1-10, “0=very dissatisfied” to “10= very satisfied”)

In case of required induction of labor for a future pregnancy, what would be your preferred method?

❐ The same method

❐ Another method

❐ Refusal of another induction of labor, whatever the device

Overall, how do you feel about your experience of childbirth (including induction of labor, labor and delivery)? ……………………………………….....

(On a visual analog scale 1-10, “0=very dissatisfied” to “10= very satisfied”)
